# Paper‐Based N‐Doped Carbon Films for Enhanced Oxygen Evolution Electrocatalysis

**DOI:** 10.1002/advs.201400015

**Published:** 2015-01-27

**Authors:** Sheng Chen, Jingjing Duan, Jinrun Ran, Shi‐Zhang Qiao

**Affiliations:** ^1^ School of Chemical EngineeringThe University of AdelaideAdelaideSA5005Australia

**Keywords:** carbon nitrate, cellulous papers, graphene, oxygen evolution reaction, synergistic effect

## Abstract

**Cellulous‐fiber papers are used as 3D structural templates** for the assembly of graphene and graphitic carbon nitrate (g‐C_3_N_4_) ultrathin nanosheets. The resultant materials, which possess highly active centers, rich porosity, and 3D conductive networks, can catalyze the oxygen evolution reaction with competitive activity and much better durability compared to the benchmark noble metal electrocatalysts (IrO_2_).

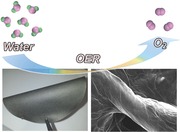

Nowadays, there are extensive interests in studying some electrochemical reactions, such as oxygen evolution reaction (OER), hydrogen evolution reaction, oxygen reduction reaction (ORR), and carbon monoxide (CO) oxidation, which are core steps in many sustainable energy techniques.^1^, ^2^, ^3^, ^4^, ^5^, ^6^, ^7^ Besides enthusiastic efforts to search for new electrocatalysts to facilitate these processes, numerous studies are also devoted to making better use of the existing electrocatalysts for achieving optimal properties.^2^, ^8^, ^9^, ^10^, ^11^ Common strategies for enhancing the performances of electrocatalysts include either optimization of their chemical compositions or micro/nanostructures.^2^, ^8^, ^9^, ^10^, ^11^ Nevertheless, these methods require complicated procedures with long‐time operation and the catalytic performances remain unsatisfactory. Recently, the concept of “support‐selective catalysis” has emerged in the literature,^5^, ^12^, ^13^ which highlights the fact that rational engineering the interfacial chemistry of supports could induce strong synergistic effects between supports and catalysts for accelerated catalysis. This concept might open up new possibilities to enhance the performance of electrocatalysts without altering their chemical compositions or micro/nanostructures. Despite great progress, the research of support‐selective electrocatalysis is still in its infant stage. Further work should be conducted to enhance the interactions between supports and electrocatalysts for improved catalytic properties.

OER is a critical step in water splitting and metal–air batteries, which involves the oxidation of water into oxygen molecules in alkaline (4OH^−^ → 2H_2_O + O_2_ + 4e^−^) or neutral/acidic solutions (2H_2_O → 4H^+^ + O_2_ + 4e^−^).^2^, ^3^, ^8^, ^9^ Because of its sluggish kinetics, OER usually proceeds on various electrocatalysts such as noble metal (irrinium oxide IrO_2_ and ruthinium oxide RuO_2_),^14^, ^15^ nonprecious metal (manganese dioxide MnO_2_,^16^ cobalt oxide Co_3_O_4_,^17^ and perovskite Ba_0.5_Sr_0.5_Co_0.8_Fe_0.2_O_3–δ_),^3^ and nonmetal materials (nitrogen‐doped graphite^18^ and carbon nanotube^11^). Commonly, two strategies have been utilized to enhance the performances of these electrocatalysts: to modify their chemical compositions by element doping^18^/hybridization,^11^ or tailor their micro/nanostructures, for example, by assembly into 1D nanowires,^10^ 2D sheets,^9^ or 3D hollow spheres.^8^ Besides these strategies, it would be also useful to tailor the interfacial chemistry of electrode supports for elevated catalytic efficiency. Nevertheless, conventional catalyst supports for OER include various nonfunctionalized substrates, for example, glassy carbon^11^ and titanium foils.^10^ Because of the general incompatibility between supports and electrocatalysts, these electrode systems only offer relatively weak interactions between each component which lead to compromised catalytic properties.

In great contrast, there is a wide range of materials with rich self‐contained groups (hydroxyl –OH, epoxyl –OCO–), such as hydrogels^19^ and cellulous fibber (CF) paper,^20^ which may constitute a new category of electrode supports (labeled as “active supports”). These supports can directly form bondings with electrocatalysts through the functional groups, resulting in strong interactions and consequently enhanced catalytic activity, kinetics, and durability. Besides, we recently demonstrated that a number of elements other than the interfacial chemistry may also significantly influence the efficiency of OER catalysis, for example, electrode architectures.^21^, ^22^ As compared to the traditional 2D planar counterparts (glassy carbon, metal foils, etc.), 3D structured electrodes with higher catalyst loadings and better electrode contact can exhibit increased catalytic activity toward OER.^21^, ^22^ In this respect, CF papers that have both large amount of functional groups (–OH and –OCO–) and 3D continuous porous framework might become an attractive target for the construction of 3D electrodes for energy‐related catalytic reactions. To our best knowledge, the reports of CF papers‐based catalytic electrodes for OER are rare.

In this work, we fabricated a 3D N‐doped carbon film by assembling graphene and graphitic carbon nitrate (g‐C_3_N_4_) ultrathin nanosheets on the frameworks of CF papers (denoted as G‐C_3_N_4_). Graphene has high electrical conductivity for fast charge transport, while g‐C_3_N_4_ ultrathin nanosheets can provide largely exposed active centers for electrocatalysis. Because of the strong synergistic effects between these components and CF papers, the resultant material can exhibit remarkable catalytic performances, with a competitive activity and much better durability as compared to the benchmark noble metal electrocatalysts for OER (IrO_2_).

G‐C_3_N_4_ was prepared according to the procedure in **Scheme** **1**. Firstly, C_3_N_4_ nanosheets (Figures S1 and S2, Supporting Information) were merged with graphene oxide (GO) to form a homogeneous light brown dispersion, which was casted onto a cellulous paper. Next, the hybrid paper was dried and then reduced with hydrazine vapor to form a black film.

**Scheme 1:**
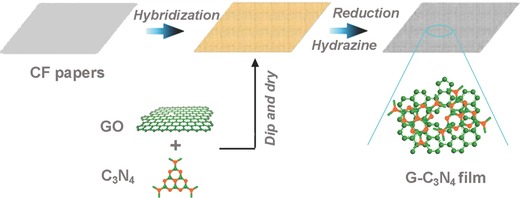
Synthetic process of G‐C_3_N_4_.

The optical image in **Figure** **1**A reveals G‐C_3_N_4_ is a flexible and shiny film of a few centimeters size and several milli­meters thick. A close examination reveals the film has rich porosity ranging from several to tens of micrometers (Figure 1B). Moreover, graphene (or reduced graphene oxide, RGO) and C_3_N_4_ nanosheets have interconnected each other to produce composited nanosheets due to their strong coupling interactions (Figures 1C,D and S3, Supporting Information). The relatively ordered assembly of graphene and C_3_N_4_ on cellulous papers with rich porosity is expected to promote efficient mass and ion transport during catalytic process.

**Figure 1:**
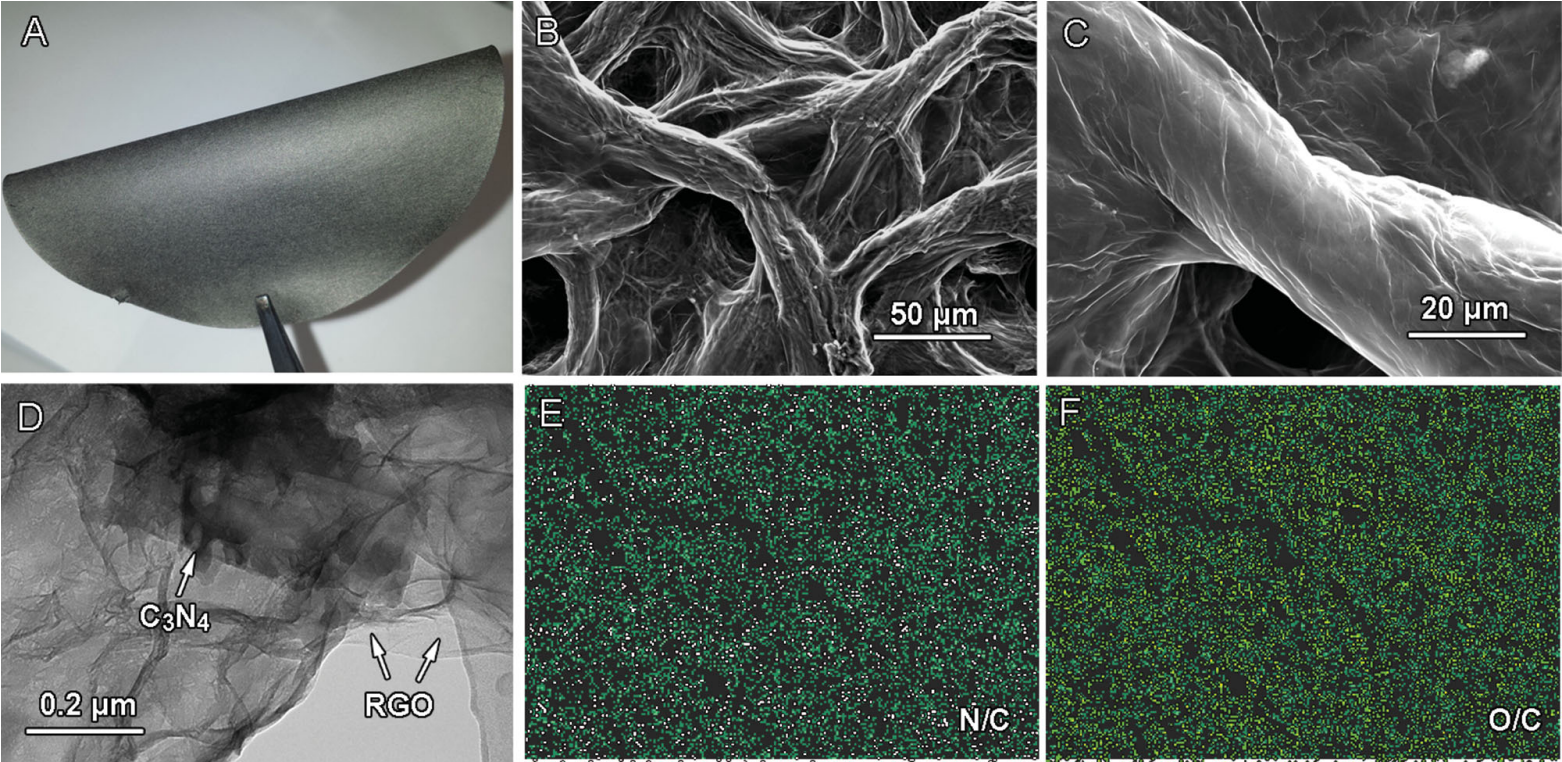
Typical morphological characterizations of G‐C_3_N_4_: A) optical image; B,C) scanning electron microscopy images; D) transission electron microscopy image; E,F) energy dispersive spectrometer element mapping taken from (C) showing distribution of N (pink), O (yellow), and C (green) atoms inside G‐C_3_N_4_.

G‐C_3_N_4_ contains C, N, and O as the main components (Figures 1E,F and S4, Supporting Information). The element mappings of selected regions show a homogeneous distribution of C, N, and O elements throughout G‐C_3_N_4_ (Figure 1E,F), indicating the uniform distribution of C_3_N_4_ and graphene sheets. Graphene and C_3_N_4_ account for 84.4 and 15.6 wt% of G‐C_3_N_4_, respectively, as calculated from thermogravimetric analysis (Figure S5, Supporting Information). Moreover, X‐ray photoelectron spectroscopy (XPS) reveals G‐C_3_N_4_ has a nitrogen/carbon atomic ratio of ≈8.8% containing both graphitic, pyrrolic and pyridinic N species, which is different from RGO with only pyridinic N species^23^ (**Figures** **2**A,B and S6, Supporting Information).

**Figure 2:**
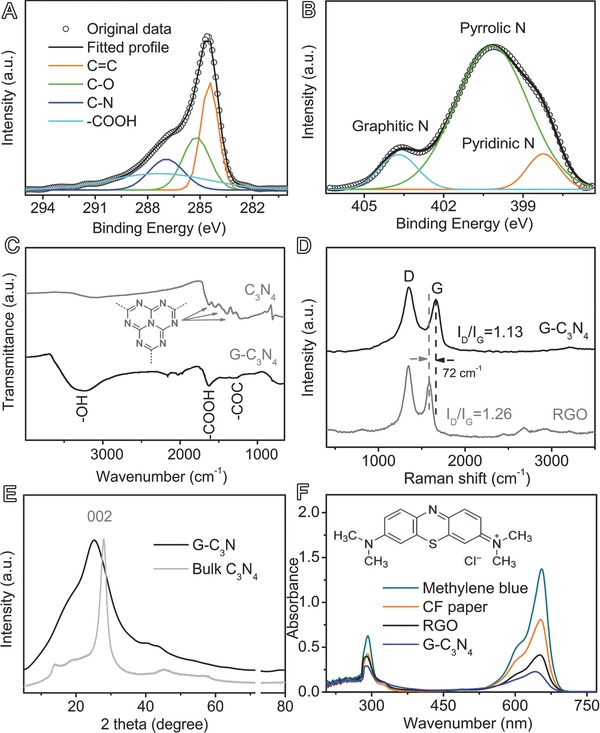
The structural analyses of G‐C_3_N_4_: A,B) XPS C1s and N1s spectrum; C) Fourier transform infrared spectra (FTIR) as compared to C_3_N_4_; D) Raman spectra as compared to graphene (RGO); E) X‐ray diffractometer (XRD) as compared to C_3_N_4_; F) UV–vis spectra as compared to other samples measured by methlylene blue experiments.

G‐C_3_N_4_ has a number of oxygen‐containing functional groups such as carboxyl (–COOH), hydroxyl (–CO), and C–N bonding (Figure 2A–C), which make it hydrophilic in nature with a contact angel of 65.3^o^ (Figure S7, Supporting Information). Meanwhile, these functional groups on graphene nanosheets also act as anchor sites for the immobilization of C_3_N_4,_ as indicated by a decrease in Raman D/G peak intensity ratio (*I*_D_/*I*_G_) of graphene in comparison to G‐C_3_N_4_ hybrid (1.26 vs 1.13, Figure 2D). The FTIR peaks around 2000 cm^−1^ for G‐C_3_N_4_ in Figure 2C stem from the C=O stretching vibration of CO_2_ impurities. The strong coupling interaction between C_3_N_4_ and graphene in hybrid film is confirmed by Raman spectra, which shows an upshift of 72 cm^−1^ in Raman G band of G‐C_3_N_4_ as compared to individual RGO (Figure 2D). Further, X‐ray diffraction (XRD) pattern of G‐C_3_N_4_ demonstrates a more broadened diffraction peak than that of C_3_N_4_ (Figure 2E), corresponding to the well‐developed porosity in the hybrid film. The surface area of G‐C_3_N_4_ is 96.4 m^2^ g^−1^ as calculated by methylene blue adsorption experiment (Figure 2F),^24^, ^25^ which is much higher than individual graphene (64.4 m^2^ g^−1^) or CF paper (3.5 m^2^ g^−1^). The high surface area of G‐C_3_N_4_ also allows easy penetration of hydrazine vapor inside hybrid film to reduce GO into graphene during the synthetic process. The hydrophilic structure and highly accessible surface of G‐C_3_N_4_ are essential for efficient oxygen evolution electrocatalysis.

The G‐C_3_N_4_ film is directly used as a working electrode to catalyze OER in alkaline electrolyte, with Ag/AgCl as a reference electrode and platinum foil as a counter electrode (**Figure** **3**A), respectively. G‐C_3_N_4_ demonstrates the highest catalytic activity as compared to other samples (Figure 3B,C). Specifically, it has a smaller onset potential of 387.2 mV than its counterparts (528.5 mV for graphene and 585.6 mV for C_3_N_4_). Its onset potential (387.2 mV) is even comparable to that of conventional noble metal benchmark for OER (IrO_2_, 373.5 mV, Figure 3C). On the other hand, the overpotential of different samples at 10.0 mA cm^−2^ (*E_i_*_=10_) was compared which is the practical operation current of solar fuel synthesis.^18^ G‐C_3_N_4_ delivers the current of 10.0 mA cm^−2^ at a relatively low overpotential of 414.5 mV as compared to all the other materials (>700 mV for both graphene and C_3_N_4_ and 510.2 mV for IrO_2_).

**Figure 3:**
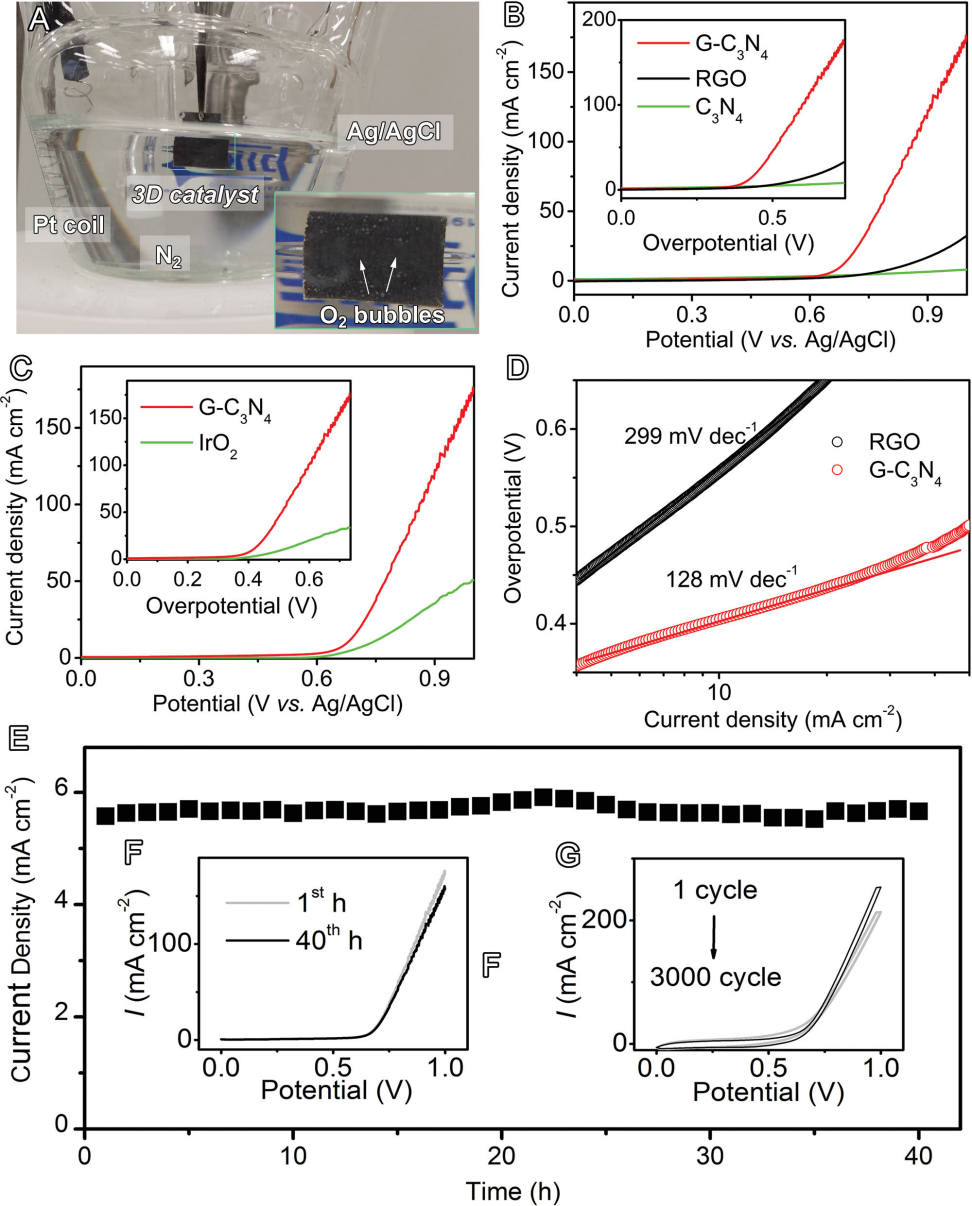
OER electrochemical catalysis on G‐C_3_N_4_ in 0.1 m KOH electrolyte: A) Optical image showing the experimental setup and oxygen bubbles on catalyst electrode (inset); B,C) linear scaning voltammograms (LSV) plots as compared to RGO, C_3_N_4_, and IrO_2_; the insets of B) and C) show the corresponding data replotted as the current density versus overpotential; D) Tafel plots as compared to RGO; E) chronoamperometric response for 40 h; F) LSV plots at the 1st and 40th h; G) cyclic voltammetry at 1st and 3000th cycles.

Moreover, the catalytic kinetics of G‐C_3_N_4_ electrode was examined by Tafel plots, which were recorded with the linear regions fitted into the Tafel equation (*η* = *b*log *j* + *a*, where *η* is overpotential, *j* is the current density, and *b* is the Tafel slope).^11^ As displayed in Figure 3D, G‐C_3_N_4_ has a lower Tafel slope of 128 mV dec^−1^ than other samples such as graphene (299 mV dec^−1^). Exchange current density (*j*_0_) was calculated by applying extrapolation method to the Tafel plots, i.e., log(|*j*_0_(mA cm^−2^)|) at *η* = 0 V. G‐C_3_N_4_ has a much higher exchange current density (79.4 μA cm^−2^) than graphene (3.2 μA cm^−2^), indicating it has a more favorable kinetics toward OER.

Further, strong durability of OER electrocatalysts is essential for its application in many energy storage and conversion devices. G‐C_3_N_4_ shows excellent stability with seldom current attenuation even after operation for 40 h (<5%, Figures 3E, and S8A,B, Supporting Information). This result is in accordance to the analysis of linear sweep voltammetry which demonstrats insignificant activity loss at 1st and 40th h (Figure 3F). Further, the high stability of G‐C_3_N_4_ electrode is confirmed by cyclic voltammetry (Figure 3G), showing only slight alternations after 3000 continuous potential cycles. The outstanding features of high activity, favorable kinetics, and strong durability indicate that G‐C_3_N_4_ is a suitable catalyst for OER.

The catalytic activity of G‐C_3_N_4_ mainly originates from C_3_N_4_ species,^11^, ^26^ while the excellent structural and interfacial properties of G‐C_3_N_4_ film contribute to significantly enhanced performance. Generally, water oxidation involves three intermediate steps, i.e., water adsorption, water dissociation and oxygen evolution. First, there is a number of the oxygen‐containing groups on G‐C_3_N_4_ such as C–O and –COOH (Figure 2A,C), which make it hydrophilic (Figure S7, Supporting Information) and thus the film can interact with water molecules via hydrogen bonding and favor adsorption of a large amount of water (approximately eight times of its own mass). These self‐contained water molecules can shift the equilibrium of water oxidation reaction (2H_2_O → 2H_2_ + O_2_) toward right side, thereby enhancing the OER catalytic kinetics. Second, the well‐developed 3D porous network of G‐C_3_N_4_ hybrid film (Figures 1B and 2E,F) permit a facile mass transport within electrodes, and the strong interactions between C_3_N_4_ and highly conductive graphene sheets could promote the electron transfer during catalytic process (Figure 2D), which consequently facilitate the maximal use of C_3_N_4_ for highly efficient water dissociation. Interestingly, the numerous active centers of G‐C_3_N_4_ can also catalyze ORR with an onset potential of −0.38 V (vs Ag/AgCl, Figure S9, Supporting Information), indicating that G‐C_3_N_4_ is a versatile catalyst for a number of catalytic processes.

Importantly, G‐C_3_N_4_ demonstrated excellent stability with insignificant performance loss after long‐term cycling (Figure 3E–G), probably due to its high mechanical flexibility and excellent structural properties. Conventional catalyst electrodes are prepared by depositing active species, such as platinum, copper, titanium, nickel, etc., on the surface of 2D or 3D metallic supports.^10^, ^11^, ^27^ Because of the general incompatibility between electrocatalysts and these supports, the active species are vulnerable to peeling off during elongated catalysis duration (Figures S10 and S11, Supporting Information). In contrast, cellulous papers have a large number of surface functional groups such as hydroxyl –OH and epoxyl –COC–. These groups can bind with the oxygen‐containing groups of reduced GO (hydroxyl C–OH, epoxyl –COC, and –COOH) to form strong forces such hydrogen bondings and covalent bondings, thereby assuring strong electrode durability during OER process.

Furthermore, nonmetal electrocatalysts have received great interest in recent years because they can combine the advantages of low cost, eco‐friendliness, and rich active centers.^11^, ^18^, ^28^ Thus far, there are only a few illustrations of nonmetallic OER electrocatalysts, for example, N(5)‐ethylflavinium,^28^ N‐doped graphite,^18^ and graphene‐CNTs hybrid.^22^ However, most of their activities are still inferior to IrO_2_. Very recently, we showed that carbon nanotubes (CNTs)/g‐C_3_N_4_ hybrid sheets could deliver a catalytic activity comparable to IrO_2_ benchmark.^11^ Nevertheless, the performance was tested by depositing active species on 2D glassy carbon electrode with a spin of 1600 rpm, thus it is not suitable for widespread applications. In this study, we demonstrate for the first time that similar catalytic activity comparable to IrO_2_ can be achieved on 3D flexible N‐doped carbon electrodes with carefully designed structural, electronic, and mechanical properties. Despite of the high activity, the 3D electrodes are merely made of very cheap and wide accessible precursors, i.e., CF papers, graphene, and C_3_N_4_. The ease of this methodology is expected to expand to the preparation of many other 3D electrocatalysts for a wide range of renewable energy harvesting, conversion, and storage devices.

In conclusion, we first introduced the concept of “support‐selective catalysis” into the research field of OER, which provide a new strategy to enhance the performances of electrocatalysts without altering their chemical compositions or microstructures. The remarkable properties of as‐synthesized G‐C_3_N_4_ material, such as highly active centers, rich porosity, and 3D conductive networks, make it an ideal candidate in electrocatalysis. Analysis of OER reveals the 3D electrode has high activity and favorable kinetics that are comparable to IrO_2_. Moreover, the catalyst electrode also shows an excellent durability during long‐term cycling due to its robust mechanical properties. This material was prepared *via* a facile “casting and drying” process with low cost precursors and may find applications for a broad range of other technological systems including metal‐air batteries, photocatalysis, supercapacitors, and heterocatalysis.

## Supporting information

As a service to our authors and readers, this journal provides supporting information supplied by the authors. Such materials are peer reviewed and may be re‐organized for online delivery, but are not copy‐edited or typeset. Technical support issues arising from supporting information (other than missing files) should be addressed to the authors.

Supplementary
