# Supplementary material for: Paper‐Based N‐Doped Carbon Films for Enhanced Oxygen Evolution Electrocatalysis
Source: Adv Sci (Weinh). 2015 Jan 27;2(1-2):1400015. doi: 10.1002/advs.201400015 (PMC5115282; doi:10.1002/advs.201400015)
Supplement: Supplementary file 1 — Supplementary [file ADVS-2-0f-s001.pdf]

Copyright WILEY-VCH Verlag GmbH & Co. KGaA, 69469 Weinheim, Germany, 2015.

Supporting Information

for *Adv. Sci.,* DOI: 10.1002/advs.201400015

Paper-Based N-Doped Carbon Films for Enhanced Oxygen Evolution Electrocatalysis

*Sheng Chen, Jingjing Duan, Jinrun Ran, and Shi-Zhang Qiao**

Supporting Information

Paper-based N-doped carbon films for enhanced oxygen evolution electrocatalysis

*Sheng Chen, Jingjing Duan, Jinrun Ran, and Shi-Zhang Qiao**

[*] Prof. Shi-Zhang Qiao, Sheng Chen, Jingjing Duan, Jinrun Ran
School of Chemical Engineering, the University of Adelaide,
Adelaide, SA 5005, Australia
E-mail: s.qiao@adelaide.edu.au

I. Experimental Section

1. Chemicals

Graphite flakes, cyanamide (CN-NH_2, 99%), potassium permanganate ($KMnO_4$, 99%), potassium persulfate ($K_2S_2O_8$, 99%), iridium(IV) oxide (IrO_2, 99%), potassium hydroxide (KOH, 99%), sulfuric acid (H_2SO_4, 95-98%), phosphorus pentoxide (P_2O_5, 98%), hydrochloric acid (HCl, 38 wt%), hydrazine hydrate ($N_2H_4 \cdot H_2O$, 80 wt%), , hydrogen peroxide (H_2O_2, 35 wt%), ethanol (EtOH, absolute), isopropanol ($(CH_3)_2$CH-OH, absolute), were purchased from Sigma-Aldrich and directly used without further treatment or purification.

2. Synthesis

Cyanamide (2 g) was calcined under an argon atmosphere at 550 ^{o}C for 6 h at a heating rate of 5 ^{o}C min^{-1} to form bulk graphitic carbon nitrate (g-C_3N_4). Then the bright yellow powder (3 mg) was mixed with isopropanol (1 mL), and subjected to bath ultrasonication for 12 h. The resultant dispersion was centrifuged at 1000 rpm for 30 min. It reveals that both single- and few-layer C_3N_4 nanosheets are produced in the top of resultant solution.[1] On the other hand, graphene oxide (GO) was prepared by chemical oxidation of natural graphite *via* the Hummer's method.[2] In a typical synthetic process of G-C_3N_4, GO dispersion (2 mg mL^{-1}, 1 mL) was mixed with C_3N_4 (1 mL), and casted onto a piece of cellulous paper. The hybrid film was dried at 50 ^{o}C for 6 h in the oven. Next, the GO and C_3N_4-coated paper was placed in an autoclave, and 500 μL hydrazine monohydrate (80 %) was added to the autoclave. After that, the autoclave was heated at 80 ^{o}C for 6 h. For the purpose of comparison, individual graphene film was prepared similarly without C_3N_4; C_3N_4 film was prepared by casting C_3N_4 solution on CF papers in a similar condition.

3. Characterization

X-ray diffractometer (XRD) measurements were performed on a Philips 1130 X-ray

diffractometer (40 kV, 25 mA, Cu Kα radiation, λ=1.5418 Å); Fourier transform infrared spectra (FTIR) were recorded on a Nicolet 6700 spectrometer; ultraviolet-visible (UV-vis) spectra were recorded on a SHIMADZU UV-2600 spectrophotometer; Raman spectra were obtained on an WiTEC alpha300R Raman microscope with a 532 nm solid laser as an excitation source; Thermogravimetric analyses (TGA) were recorded on a TGA/SETARAM thermogravimetric analyzer from 100 to 500 ^{o}C with a heating rate of 10 $^{o}C\cdot min^{-1}$ in O_2 flow; the contact angle was measured on a Theta/Attension Optical Tensiometer; X-ray photoelectron spectra (XPS) were performed on an Axis Ultra (Kratos Analytical, UK) XPS spectrometer equipped with an Al Ka source (1486.6 eV); morphologies of the samples were observed on transmission electron miscropy (TEM, Tecnai G2 Spirit) and scanning electron microscopu (SEM, QUANTA 450); Energy Dispersive Spectrometer (EDS) and elemental mapping patterns were acquired on the SEM (QUANTA 4500). Moreover, methylene blue (MB) adsorption experiments were used to probe the accessible surface area of the samples.[3, 4] Specifically, the samples were added into a MB-containing ethanol solution (0.1 mg ml^{-1}) and were left at 25 ^{o}C for 48 h to allow the accessible surface of the samples to be covered by MB molecules. The amount of the adsorbed MB was calculated by using the change in the MB concentration measured by using UV-Vis spectroscopy. The surface area was estimated under the assumption that each milligram of adsorbed MB occupies 1.35 m^2 of the surface.[3, 4]

4. Electrochemical testing

Electrochemical measurements were conducted in a standard three-electrode glass cell on a 760 workstation (Pine Research Instruments, US) using hybrid films as working electrodes, platnium wire as a counter electrode and Ag/AgCl/KCl (3 M) as a reference electrode (please see the testing devices in supplementary results). The mass density of G-C_3N_4 was ~0.1 mg cm^{-2}. The electrolyte was prepared using Milli-Q water (18 M Ω cm^{-1}) and KOH. OER data were collected under N_2 (ultra-high-grade purity, Airgas) LSV and CV were

recorded with the scan rates in the range of 5~100 mV s^{-1}; the current density was normalized to the geometrical area; the measured potentials *vs.* Ag/AgCl were converted to a reversible hydrogen electrode (RHE) scale according to the Nernst equation ($E_{RHE} = E_{Ag/AgCl} + 0.059$ pH + 0.197).[5, 6] The overpotential was obtained according to the equation: $\eta = E_{RHE} - 1.23$ V. Tafel slope was calculated according to the Tafel equation: $\eta = b\log j + a$, where η is overpotential (V), j is current density (mA cm^{-2}), and b is Tafel slope (mV dec^{-1}).Exchange current density (j_0, mA cm^{-2}) was calculated by applying extrapolation method to the Tafel plots, *i.e.*, $\log(|j_0(\text{mA cm}^{-2})|)$ at η=0 V.

Moreover, analysis of ORR has been conducted in a similar three-electrode system in 0.1 M KOH electrolyte. A 30 mL $min^{-1}$of O_2 flows into the electrolyte during the collection of electrochemical data in order to ensure the O_2/H_2O equilibrium at 1.23V *vs.* RHE. LSV plot was collected at 5 mV s^{-1} from 0.1 V to -0.8 V (*vs.* Ag/AgCl).

For the purpose of comparison, the catalytic performance of IrO_2 powder was tested on a glassy carbon working electrode under similar conditions. The working electrode was prepared by the drop casting method. Specifically, IrO_2 (2 mg) was ultrasonically dispersed in Milli-Q water (1 mL) with Nafion (30 μL of 0.1%), and then the catalyst dispersion was transferred onto the glassy carbon electrode and dried (catalyst loading ~0.1 mg cm^{-2} for IrO_2).

II. Supplementary Results

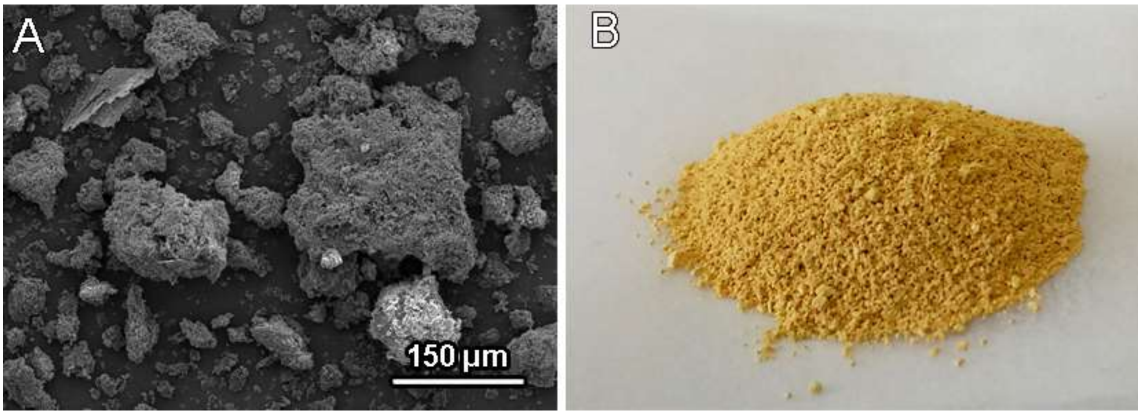

Figure S1. Structural characterizations of bulk C_3N_4: (A) SEM image; (B) Optical image.

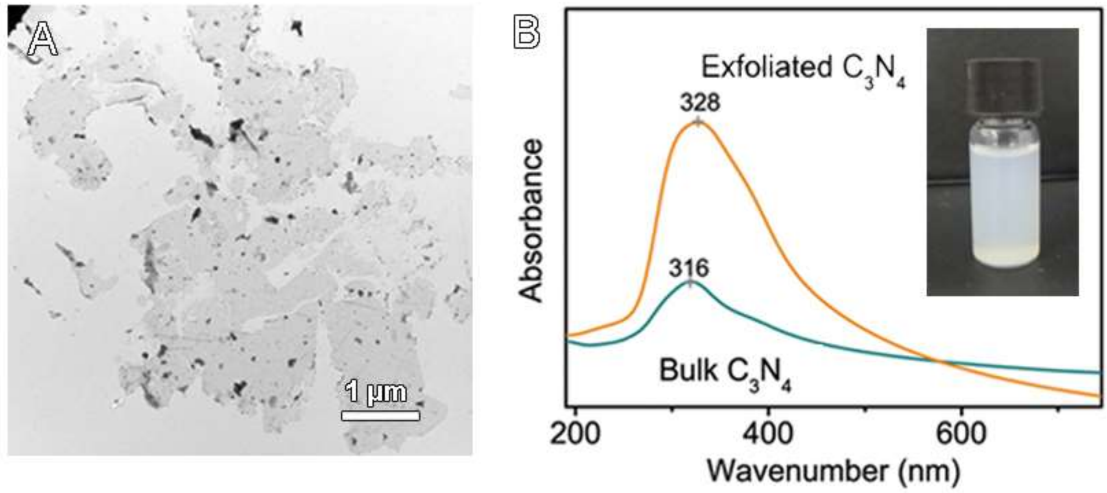

Figure S2. (A) SEM image of exfoliated C_3N_4 nanosheets: (B) UV-vis spectra of exfoliated C_3N_4 nanosheets in comparison to their bulk counterpart; (C) Optical image of exfoliated C_3N_4 nanosheets.

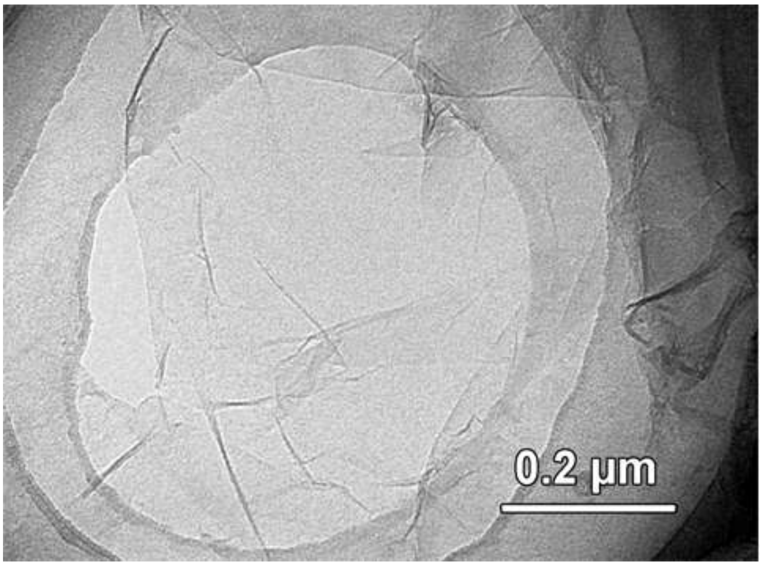

Figure S3. TEM image of graphene film.

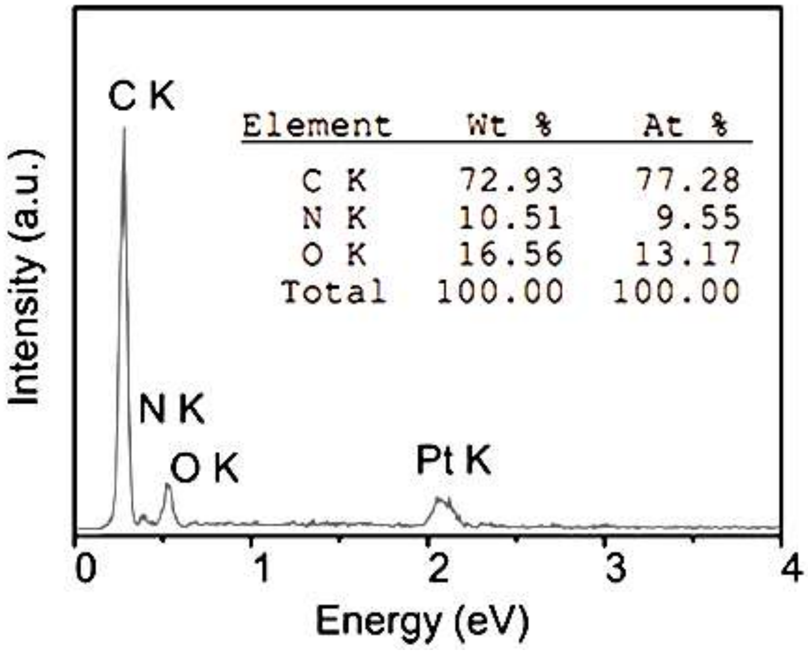

Figure S4. EDS analysis of G-C_3N_4 film.

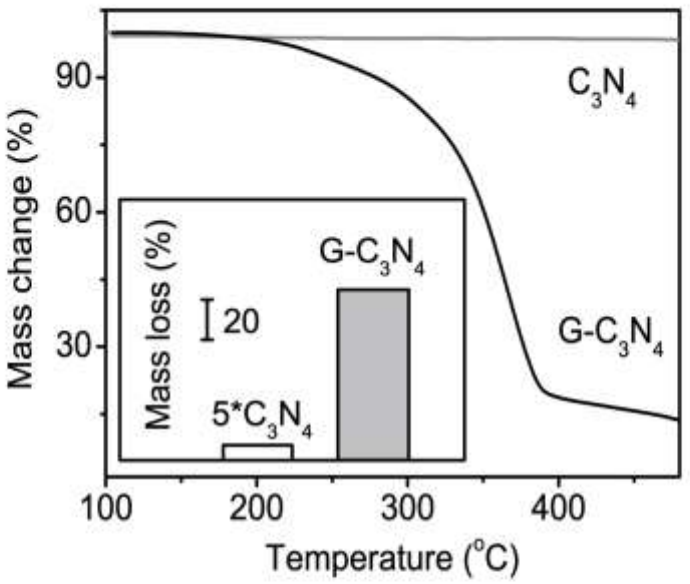

Figure S5. TGA analysis of G-C_3N_4 in comparison to C_3N_4

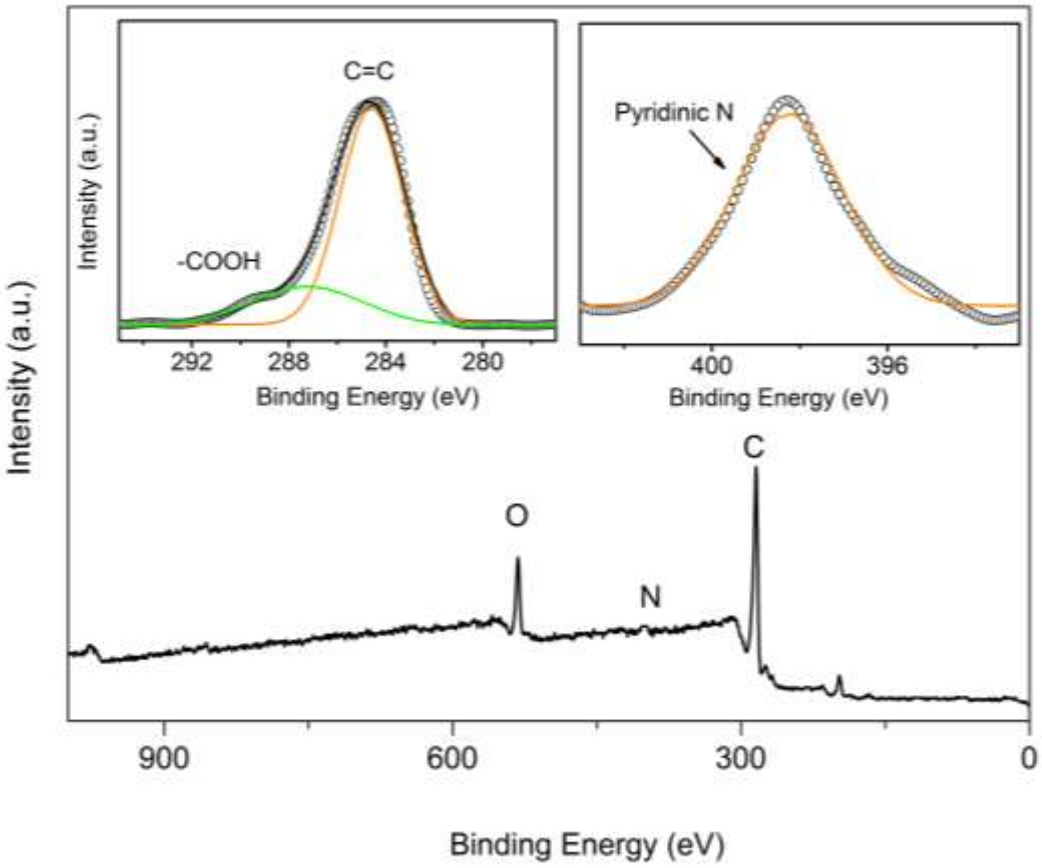

Figure S6. XPS analyses of graphene film.

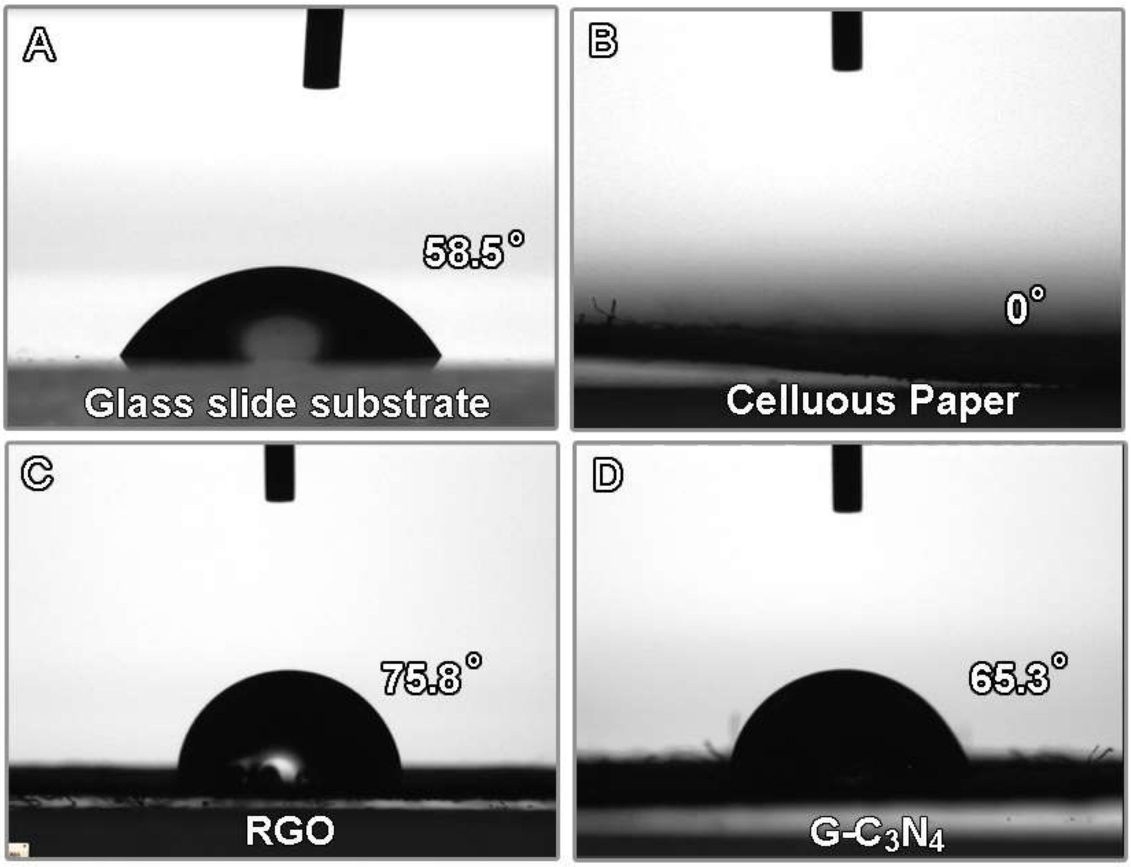

Figure S7. Contact angles of different samples: (A) Glass slider substrate; (B) Cellulous Paper; (C) graphene (RGO); (D) G-C_3N_4.

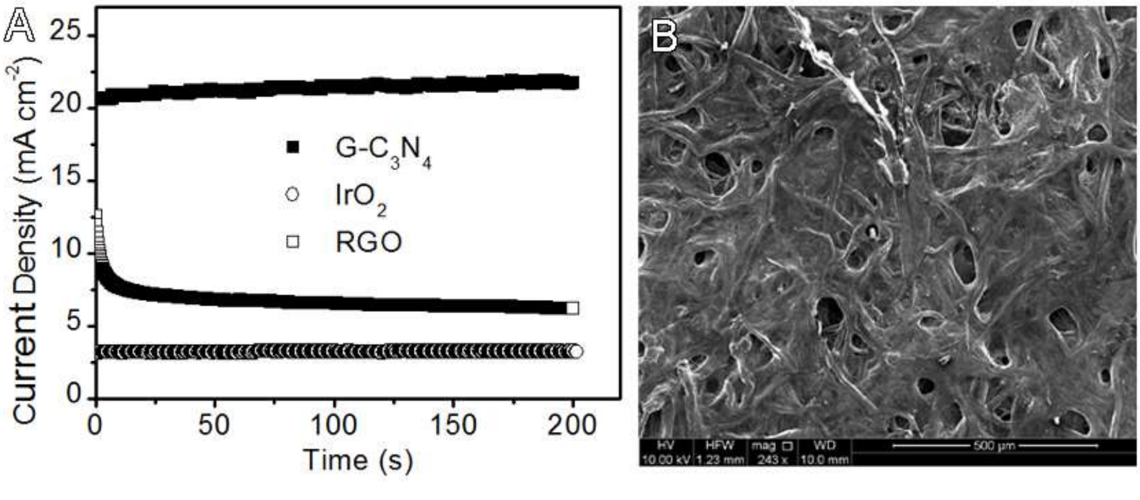

Figure S8. (**A**) Chronometric response of G-C_3N_4 in comparison to RGO and IrO_2; (B) SEM image of G-C_3N_4 after cycling operation for 40 h.

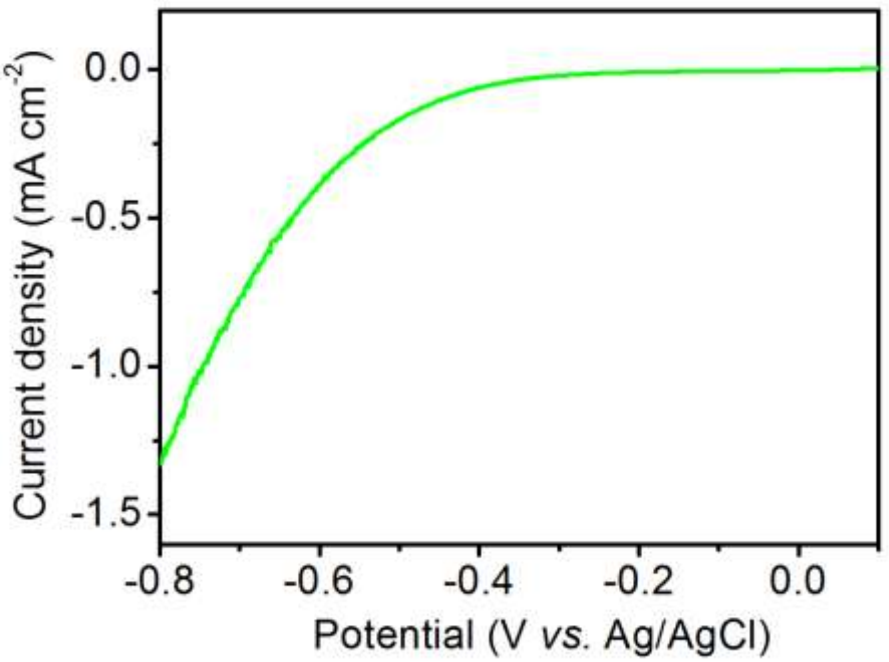

Figure S9. Electrochemical study of G-C_3N_4 in 0.1 M KOH for electrocatalytic oxygen reduction reaction (ORR).

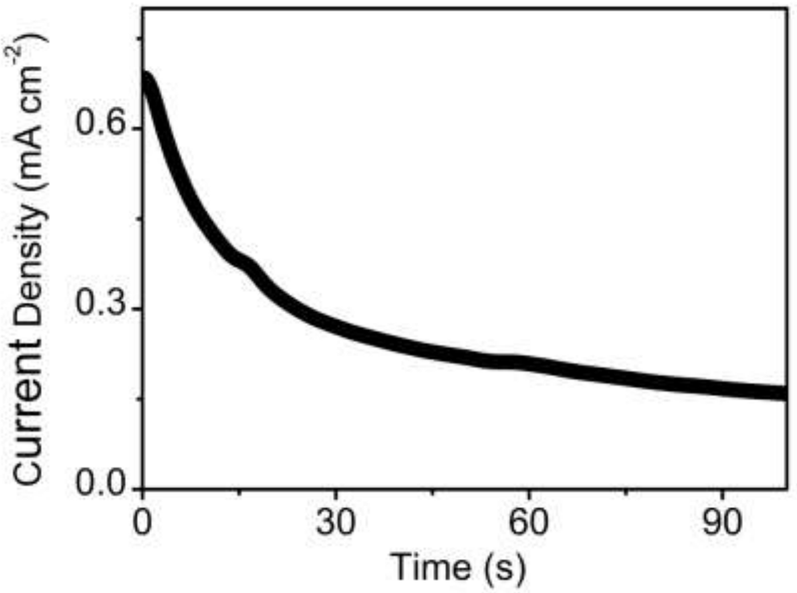

Figure S10. Chronometric response of G-C_3N_4 on glassy carbon electrode.

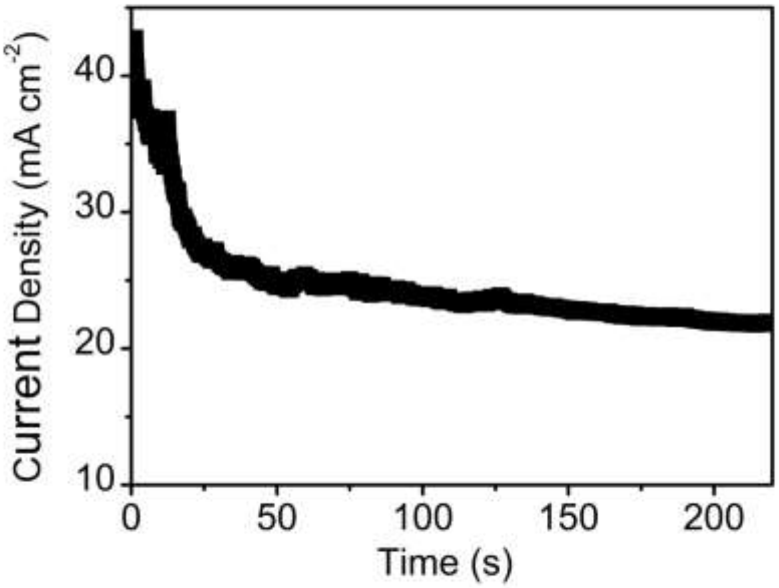

Figure S11. Chronometric response of G-C_3N_4 on nickel foam substrate.

References

[1] S. Yang, Y. Gong, J. Zhang, L. Zhan, L. Ma, Z. Fang, R. Vajtai, X. Wang, P. M. Ajayan, *Adv. Mater.* **2013**, *25*, 2452.

[2] W. S. Hummers, R. E. Offeman, *J. Am. Chem. Soc.* **1958**, *80*, 1339.

[3] M. F. El-Kady, V. Strong, S. Dubin, R. B. Kaner, *Science* **2012**, *335*, 1326.

[4] P. T. Hang, G. Brindley, *Clays Clay Miner.* **1970**, *18*, 203.

[5] T. Y. Ma, S. Dai, M. Jaroniec, S. Z. Qiao, *Angew. Chem. Int. Ed.* **2014**, *53*, 7281.

[6] Y. Zhao, R. Nakamura, K. Kamiya, S. Nakanishi, K. Hashimoto, *Nat. Commun.* **2013**, *4*, 2390.
